# Process of Disinfection

**Published:** 1866-06

**Authors:** 


### PROCESS OF DISINFECTION.

A memorandum on disinfection has been issued by the Privy Council, (Great Britain). In view of the approaching epidemics, we give its main points, after the *Chemical News and Druggists' Circular:*—

"1. For artificial disinfection, the agents most useful are—chloride of lime, quicklime, and Condy's manganic compounds. Metalic salts—perchloride of iron, sulphate of iron, and chloride of zinc, are applicable. In certain cases chlorine gas or sulphurous acid gas may be used; and in other cases powdered charcoal or fresh earth.

"2. If perchloride of iron or chloride of zinc be used, the common concentrated solution may be diluted with eight or ten times its bulk of water. Sulphate of iron or chloride of iron may be used in the proportion of a pound to a gallon of water, taking care that the water completely dissolves the sulphate of iron, or has the chloride of lime thoroughly mixed with it. Condy's stronger fluid (red) may be diluted with fifty times its bulk of water; his weaker fluid (green) with thirty times its bulk of water. When the matters requiring to be disinfected are matters having an offensive smell, the disinfectant should be used till this smell has entirely ceased.

"3. In the ordinary emptying of privies or cesspools, use may be made of perchloride of iron, or chloride of zinc, or of sulphate of iron. But where disease is present, it is best to use chloride of lime or Condy's fluid. Where it is desirable to disinfect, before throwing away the evacuations from the bowels of persons suffering from certain diseases, the disinfectant should be put into the night-stool or bed-pan when about to be used by the patient.

"4. Heaps of manure, or of other filth, if it be impossible or inexpedient to remove them, should be covered to the depth of two or three inches with a layer of freshly burnt vegetable charcoal in powder. Freshly burnt lime may be used in the same way, but is less effective than charcoal. If neither charcoal nor lime be at hand, the filth should be covered with a layer of some inches thick of clean dry earth.

"5. Earth near dwellings, if it has become offensive or foul by the soakage of decaying animal or vegetable matter, should be treated on the same plan.

"6. Drains and ditches are best treated with chloride of lime, or Condy's fluid, or with perchoride of iron. A pound of good chloride of lime will generally well suffice to disinfect 1000 gallons of running sewerage; but, of course, the quantity of disinfectant required will depend upon the amount of filth in the fluid to be disinfected.

"7. Linen and washing apparel requiring to be disinfected should without delay be set to soak in water containing per gallon about an ounce either of chloride of lime or Condy's red fluid. The latter, as not being corrosive, is preferable. Or the articles in question may be plunged at once into boiling water, and afterward, when at wash, be actually boiled in the washing water.

"8. Woollens, bedding, or clothing which cannot be washed, may be disinfected by exposure for two or more hours in chambers constructed for the purpose to a temperature of 210 to 250 degrees Fahrenheit.

"9. For the disinfection of interiors of houses, the ceilings and walls should be washed with quicklime water. The woodwork should be cleansed with soap and water, and subsequently washed with a solution of chloride of lime, about two ounces to the gallon.

"10. A room, no longer occupied, may be disinfected by sulphurous acid gas or chlorine gas—the first by burning in the room an ounce or two of flowers of sulphur in a pipkin; the second by setting in the room a dish containing a-quarter of a pound of finely-powdered black oxyd of manganese, over which is poured half a pint of muriatic acid, previously mixed with a-quarter of a pint of water. In either case, the doors, chimney, and windows of the room must be kept carefully closed during the process, which lasts for several hours."

